# High contributions of sea ice derived carbon in polar bear (*Ursus maritimus*) tissue

**DOI:** 10.1371/journal.pone.0191631

**Published:** 2018-01-23

**Authors:** Thomas A. Brown, Melissa P. Galicia, Gregory W. Thiemann, Simon T. Belt, David J. Yurkowski, Markus G. Dyck

**Affiliations:** 1 Marine Ecology and Chemistry, Scottish Association for Marine Science, Oban, United Kingdom; 2 School of Geography, Earth and Environmental Sciences, University of Plymouth, Plymouth, United Kingdom; 3 Department of Biology, York University, Toronto, Ontario, Canada; 4 Faculty of Environmental Studies, York University, Toronto, Ontario, Canada; 5 Department of Biological Sciences, University of Manitoba, Winnipeg, Manitoba, Canada; 6 Wildlife Research Section, Department of Environment, Government of Nunavut, Igloolik, Nunavut, Canada; University of Alberta, CANADA

## Abstract

Polar bears (*Ursus maritimus*) rely upon Arctic sea ice as a physical habitat. Consequently, conservation assessments of polar bears identify the ongoing reduction in sea ice to represent a significant threat to their survival. However, the additional role of sea ice as a potential, indirect, source of energy to bears has been overlooked. Here we used the highly branched isoprenoid lipid biomarker-based index (H-Print) approach in combination with quantitative fatty acid signature analysis to show that sympagic (sea ice-associated), rather than pelagic, carbon contributions dominated the marine component of polar bear diet (72–100%; 99% CI, *n* = 55), irrespective of differences in diet composition. The lowest mean estimates of sympagic carbon were found in Baffin Bay bears, which were also exposed to the most rapidly increasing open water season. Therefore, our data illustrate that for future Arctic ecosystems that are likely to be characterised by reduced sea ice cover, polar bears will not only be impacted by a change in their physical habitat, but also potentially in the supply of energy to the ecosystems upon which they depend. This data represents the first quantifiable baseline that is critical for the assessment of likely ongoing changes in energy supply to Arctic predators as we move into an increasingly uncertain future for polar ecosystems.

## Introduction

Polar bears (*Ursus maritimus*) are an ice-obligate species, utilising sea ice for hunting, travelling and mating[1]. Accordingly, the recent decline in Arctic sea ice extent[2] is likely to be a serious threat to polar bears[3] as recognised by the IUCN[4]. Recent simulations have estimated a 71% probability that the mean global population of polar bears will decrease by > 30% over the next 3–4 decades if sea ice continues to decline at its current rate[3]. However, such assessments are based mainly on the value of sea ice as a physical habitat and its influence on, for example, seasonal sea ice-terrestrial migratory movement [5], hunting and feeding success [6], habitat availability for denning [7] and cub survival effects [8] and so likely underestimate the additional potential value of sea ice as an underlying energy source to the food web upon which polar bears prey.

Annually, sympagic (sea ice associated) algae are estimated to provide a substantial proportion of marine energy, ranging from 3 to 57% of total Arctic primary production [9, 10] and can, during spring, represent up to as much as 100% of the algal primary production in surface waters[11]. The importance of this ice algae to the base of the Arctic ecosystem has been quantified previously using stable carbon and nitrogen isotopes, fatty acids and highly branched isoprenoid (HBI) lipids to demonstrate that the dietary content of some zooplankton can be as high as 100% sympagic algae[12–14]. However, the challenges associated with tracing and quantifying sympagic carbon through higher trophic levels of Arctic food webs, experimentally, is challenging, which likely explains a general paucity of such quantitative sympagic carbon data for polar bears in the literature.

**Fig 1:**
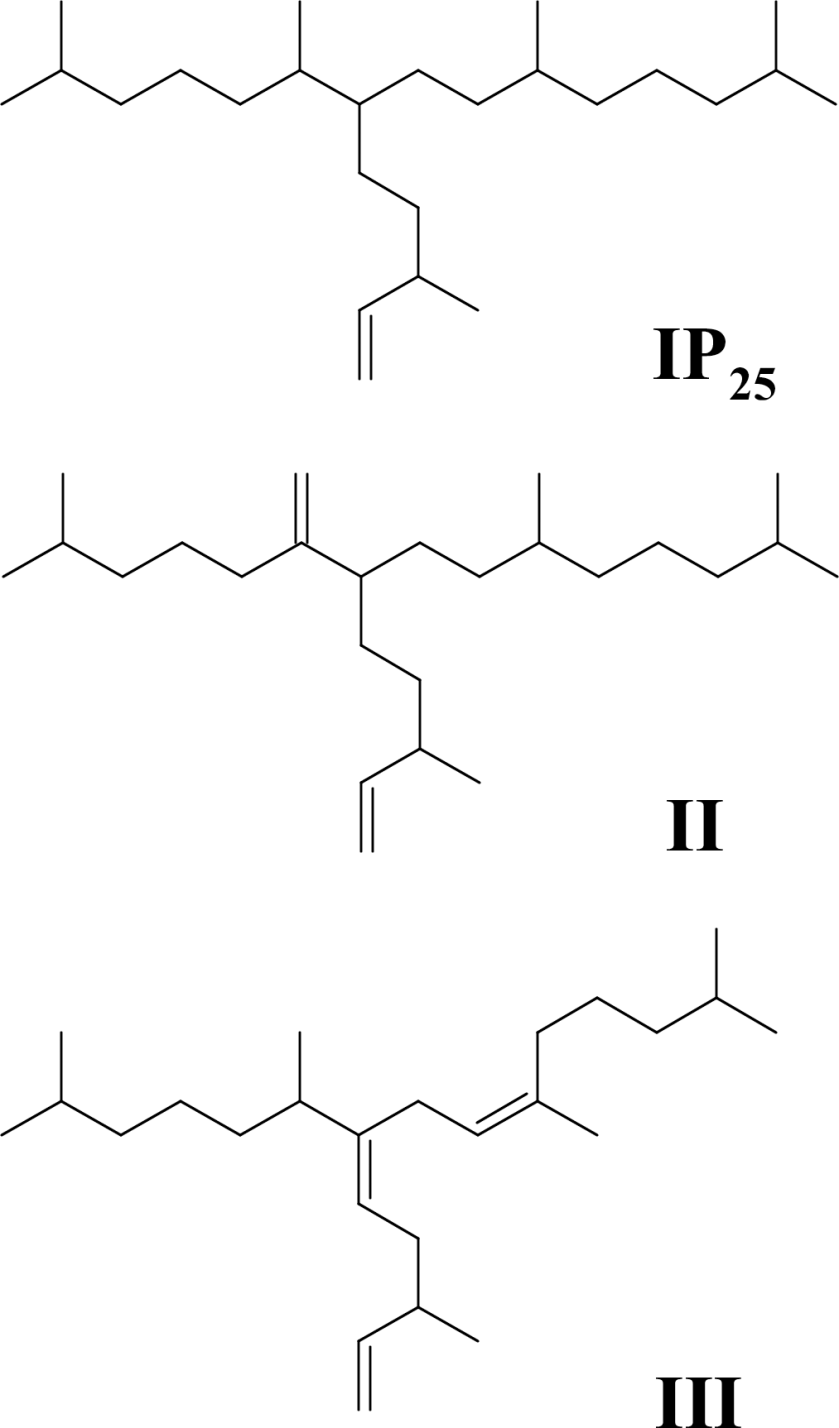
Structures of highly branched isoprenoid lipids. Structures of C_25_ highly branched isoprenoid lipids measured in polar bear liver for calculation of quantitative H-Prints.

Recently, the analysis of certain source-specific diatom lipid biomarkers (HBIs) has begun to provide quantitative estimates of sympagic carbon in the Arctic. First identified in the marine diatoms *Haslea ostrearia* and *Rhizosolenia setigera* [15], HBIs have subsequently been reported in a number of other marine diatom genera including *Pleurosigma* and *Berkeleya*[16, 17]. Some of these diatom sources represent common components of sympagic algae and produce one mono-unsaturated HBI that has not been identified in any pelagic species. Thus, the Arctic sea ice diatoms *H*. *crucigeroides*, *H*. *spicula*, *H*. *kjellmanii* and *P*. *stuxbergii* var *rhomboides* biosynthesise a highly source-specific HBI which has become known as the “Ice Proxy with 25 carbon atoms” or “IP_25_” (Fig 1[18, 19]). Following the initial detection of IP_25_ within Arctic animals[20], direct (albeit qualitative) links were subsequently observed between sympagic algae and Arctic consumers[21]. In an effort to quantify the composition of sympagic algae carbon consumed, analysis of IP_25_ in animals were supplemented with further HBIs that are common within pelagic diatoms (e.g. III; Fig 1). Recently the planktonic species *R*. *setigera*, *R*. *polydactyla f*. *polydactyla* and *R*. *hebetata f*. *semispina* were identified as *in-situ* sources of III in polar and sub-polar marine settings[22], with *Pleurosigma intermedium* also producing III in culture[23]. In fact, III is the most widely reported HBI in marine sediments worldwide[24], supporting its common production by certain phytoplanktic marine diatoms. By combining the relative abundances of HBIs of both sea ice and planktonic algae origin (Eq 1), a unique HBI-fingerprint, or “H-Print”, was initially proposed as an indicator of the relative composition of sympagic (H-Print = 0%) and pelagic (H-Print = 100%) algae in a given sample.

$$H-Print\;(\%)=\frac{\left(pelagic\;HBIs\right)}{\left(sympagic\;HBIs+pelagic\;HBIs\right)}\times 100$$ Eq 1

Although other HBIs have been reported in the marine environment[25], it was recently found that IP_25_ and II best represented sympagic algae, while III most clearly represented phytoplankton[25]. Using these three HBIs, the ability of the H-Print to accurately reflect mixed-source compositions of algae was determined by calibration, following analysis of known quantities of sympagic and pelagic algae[25]. From this calibration, a linear model was constructed to enable quantitative estimates of the proportion of sea ice vs. phytoplanktic algae carbon in animals. Subsequent tests demonstrated that the original source H-Print was transferred into the food web[25]. Accordingly, analysis of the H-Print has the potential to provide the quantitative data required to assess the proportion of sympagic algae carbon reaching higher trophic levels[12].

Here we apply the H-Print approach to samples of polar bear liver to obtain quantitative estimates of the proportions of sympagic and pelagic marine carbon reaching these top predators. Based on the established reliance of bears on sea ice as a physical platform for hunting[1], we anticipated that the ecosystem upon which the bears were dependent for prey would contain higher proportions of sea ice carbon and that this would be reflected in bears with relatively low H-Prints.

## Materials and methods

### Sample collection

**Fig 2:**
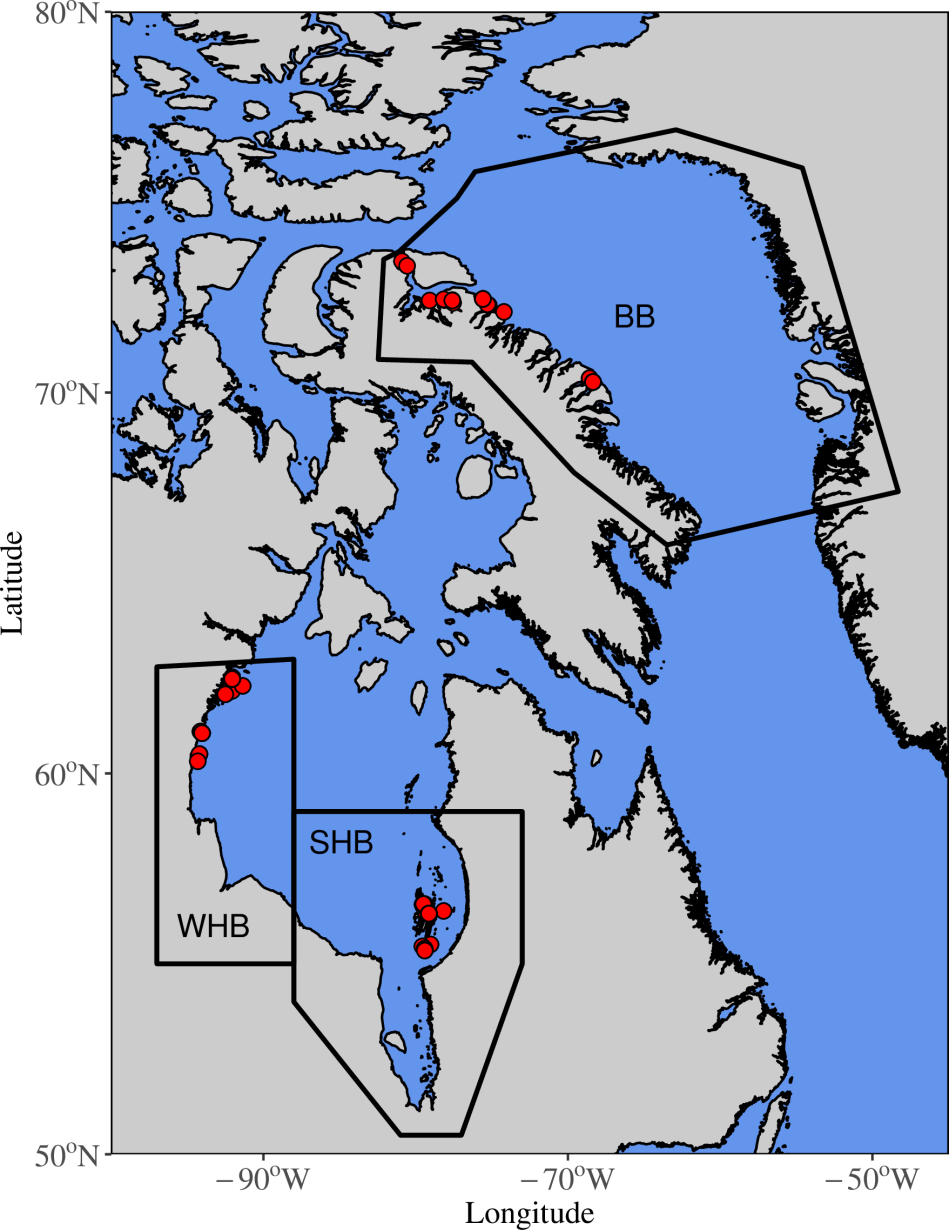
Geographic setting. Map of polar bear subpopulations and locations of harvest (red dots) in Baffin Bay (BB), western Hudson Bay (WH) and southern Hudson Bay (SH). Coastlines were created using the Global Self-consistent, Hierarchical, High-resolution Geography database distributed under the GNU Lesser General Public license [28].

Polar bear samples were collected by Inuit hunters during annual subsistence hunts and were harvested adhering to local guidelines, following territorial acts and regulations; samples were collected under approved Wildlife Research Permits 2012–026, 2013–018, and 2014–006. We analysed freeze-dried liver (HBIs and δ^15^N) and adipose tissue (fatty acids) from 55 individual polar bears (Fig 2) between October-May (2012–14) (S1 Table). Liver was chosen for HBI and δ^15^N analysis because it is metabolically highly active resulting in turnover times on the order of weeks to 1 month[26]. This was demonstrated previously where H-Print analysis of >300 ringed seals (*Pusa hispida*) enabled a seasonal scale assessment of diet in Cumberland Sound[27].

### H-Print

Liver tissue (0.4–2.4 g) was saponified (~ 5 mL H_2_O:MeOH, 1:9; 20% KOH; 60 mins; 70°C) and mixed with hexane (3 x 4 mL), then centrifuged (2 min; 2500 revolutions per minute), with hexane then being transferred and dried (N_2_ stream). Dried lipid extracts were fractionated (5 mL hexane) using column chromatography (SiO_2_; 0.5 g). HBIs were analysed by gas chromatography–mass spectrometry and quantified by measuring the mass spectral intensities for each HBI in selective ion monitoring (SIM) mode[29]. The H-Print was calculated using the analytical intensities of three HBIs (IP_25_: *m/z* 350.3, II; *m/z* 348.3 and III; *m/z* 346.3), according to Eq 2, since this combination enabled a linear calibration to be constructed previously[25].

$$H-Print\;(\%)=\frac{\left(III\right)}{\left({IP}_{25}+II+III\right)}\times 100$$ Eq 2

### Sympagic carbon estimates

Sympagic carbon, as a proportion of marine-origin carbon within polar bear livers, was estimated using Eq 3 from previous H-Print calibration (R^2^ = 0.97, *P* = <0.01, df = 23[25]). Sympagic carbon estimates are expressed here as mean values with the 99% confidence interval of estimates in parenthesis.

Sympagiccarbon%=101.08−1.02×H−Print Eq 3

### Quantitative fatty acid signature analysis (QFASA)

Fatty acids were extracted with CHCl_3_, MeOH and H_2_O [30] and derivatised to methyl esters using MeOH and H_2_SO_4_[31] and analyzed using gas-liquid chromatography—flame-ionization detection[31]. Diet composition of each bear was estimated via quantitative fatty acid signature analysis, QFASA[32], by modelling fatty acid (FA) profiles or “signatures” of bears as a linear combination of mean prey signatures. Our prey dataset included 6 prey species available in all 3 regions. Prey were collected during the annual subsistence harvest in Baffin Bay, western Hudson Bay, southern Hudson Bay, and adjacent polar bear subpopulations from 2003 to 2012. When available, prey samples from a given polar bear subpopulation were used to model polar bear diet from that same subpopulation. In cases when prey samples were not available for a given region, samples were used from adjacent/nearby regions. For Baffin Bay, prey libraries are detailed in Galicia et al[33] and are comprised of bearded seal (*Erignathus barbatus*; Davis Strait, Foxe Basin and Western Hudson Bay), beluga whale (*Delphinapterus leucas*; Davis Strait, Lancaster Sound, Northern Beauport Sea, Southern Beaufort Sea, Southern Hudson Bay and Western Hudson Bay), harbour seal (*Phoca vitulina*; Western Hudson Bay), harp seal (*Pagophilus groenlandicus*; Davis Strait), ringed seal (Lancaster Sound) and walrus (*Odobenus rosmarus*; Foxe Basin and Lancaster Sound). Narwhal (*Monodon monoceros;* Baffin Bay and Lancaster Sound) was included in the model for Baffin Bay, but was absent from bear diet composition. For southern Hudson Bay and western Hudson Bay, prey libraries were combined, comprising bearded seal (Western Hudson Bay), beluga whale (Western Hudson Bay and Southern Hudson Bay), harbour seal (Western Hudson Bay), harp seal (Davis Strait), ringed seal (Western Hudson Bay) and walrus (Foxe Basin). Diet simulations conducted in previous studies[33, 34] indicated that differences in fatty acid composition among these prey species were greater than spatial differences within prey species, and thus pooling prey samples increases sample size and improved model performance. Polar bear diets were estimated as the proportional combination of prey that minimized statistical distance between observed and modelled predator FA signatures, after accounting for patterns of lipid metabolism[32, 35]. Modelling procedures are described elsewhere[34].

### Nitrogen stable isotopes

Prior to stable nitrogen isotope (δ^15^N) analysis on polar bear liver samples, lipids were removed using a 2: 1 CHCl_3_:MeOH solvent following a modified Bligh and Dyer method[36]. Between 400–600 μg lipid extracted liver tissue was weighed into tin capsules where δ^15^N values were measured by a Thermo Finnigan DeltaPlus mass-spectrometer coupled with an elemental analyzer[29]. Analytical precision, (SD of replicate analyses; NIST 1577c, n = 7; NIST 8414, n = 46; tilapia muscle, n = 53), was ≤ 0.1*‰* Instrumental accuracy (NIST 8573 and 8547, n = 19) was ≤ 0.1*‰*.

### Numerical analysis

Numerical analyses were undertaken in R (version 3.3.2). QFASA diet estimates were done using the “qfasar” package (version 1.2.0)[37]. Conversion of H-Prints into sympagic carbon estimates were done using the previously defined regression model; Eq 3[25]. The Kruskal-Wallis test was used to compare H-Prints, δ^15^N and QFASA-derived diets between sample variables (population, sex, age and harvest date) since it does not assume data normality and accepts groups of different sizes (homoscedasticity was confirmed using Bartlett’s test at *P* = 0.05). Where significant differences were identified, Nemenyi’s post-hoc test (corrected for ties) was used to carry out pairwise comparisons to identify significantly different factors. Statistical tests were considered significant at α = 0.05.

## Results

**Fig 3:**
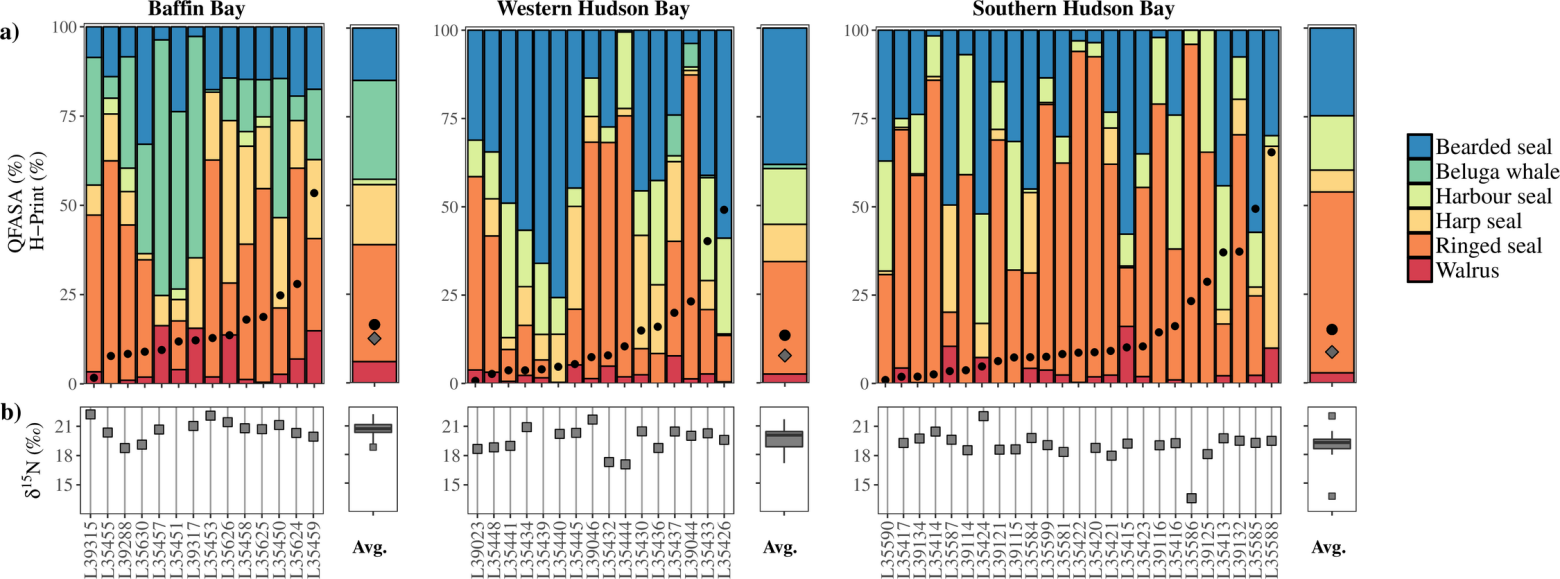
Polar bear (*Ursus maritimus*) data. a) QFASA estimates of marine mammal prey (Bearded seal *(Erignathus barbatus*), beluga whale (*Delphinapterus leucas*), harbour seal (*Phoca vitulina*), harp seal (*Pagophilus groenlandicus*), ringed seal (*Pusa hispida*) and walrus (*Odobenus rosmarus*)) consumed by individual polar bears (stacked coloured bars) and overlaid with H-Prints (black circles) of individual bears. Individual polar bears are grouped according to the geographical location of collection and the corresponding subpopulation designation: Baffin Bay, western Hudson Bay and southern Hudson Bay (see Fig 2). For each subpopulation, mean QFASA estimates of marine mammal prey and mean (black circles) and median (grey diamonds) H-Prints are summarised in the single plot adjacent to each subpopulation plot (for H-Print-derived estimates of sympagic carbon, refer to Table 1). b) δ^15^N of individual bears (grey squares). For each subpopulation, mean δ^15^N are summarised in the single plot (box and whiskers) adjacent to each subpopulation plot.

Each of the HBIs were quantifiable within all 55 liver samples, including bears of both sexes and all ages from each year and population sampled (Fig 3). Low H-Prints (<10%), indicative of mainly sympagic carbon contributions, were recorded for every month, accounting for 53% of all bears sampled. Only two bears had H-Prints > 50% (December 2012 and February 2013). None of the tested variables were significant predictors of polar bear H-Prints including; sample months (H(5) = 9.8, *P* = 0.08), population (H(2) = 2.4, *P* = 0.3), bear sex (H(1) = 0.3, *P* = 0.8), bear age (H(1) = 1.9, *P* = 0.2) or estimated prey species (*P* = >0.4). Conversion of H-Prints to estimates of sympagic carbon, using Eq 3, indicated that, on average 86% (72–100; 99% CI) of the marine carbon reaching polar bears was of sympagic origin (Table 1).

**Table 1:** Summary data for polar bears studied.

Population	Biometrics			Sympagic carbon (%)			Sea ice melt onset (days decade^-1^; 1979–2014)[2]	Interval between spring ice melt and autumn freeze (days decade^-1^; 1979–2014)[2]
	Age yrs (5+: 3–4)	Sex (M:F)	Years sampled (2012:2013:2014)	Mean	Minimum	maximum		
**Baffin Bay**	20:13	18:7	7:10:8	82 (68–96)	47 (33–61)	100 (85–114)	-7.3	+12.7
**Western Hudson Bay**	20:9	25:4	16:13:0	88 (74–102)	45 (31–59)	100 (86–115)	-5.1	+8.7
**Southern Hudson Bay**	21:13	28:6	0:20:14	87 (73–101)	35 (20–48)	100 (86–115)	-3.0	+6.6
**all bears**	61:35	71:17	23:43:22	86* (72–100)	-	-	-	-

*Calculated using the mean of the three sub-populations

Biometric data with sympagic carbon estimates (%), calculated from H-Prints using Eq 3 (99% CI[25]), of bear diet with regional sea ice metrics.

In contrast to H-Prints, mean δ^15^N values differed regionally (H(2) = 13.5, *P* = 0.001) with Baffin Bay bears being generally higher than those from southern (*P* = <0.001) and western (*P* = 0.04) Hudson Bay. Neither bear age (H(1) = 3.5, *P* = 0.06), or sex (H(1) = 0.07, *P* = 0.8) influenced the δ^15^N of bears for any of the three population in this study. QFASA indicated highly variable diet compositions between individual bears with significant regional differences in some prey (Table 2). For example, together, bearded seals and ringed seals comprised 47%, 70% and 76% of mean bear prey in Baffin Bay, western Hudson Bay and southern Hudson Bay respectively (Fig 3). In contrast, the average contribution of beluga whale, as a component of bear diet, was 28%, 1% and 0% from the same three locations.

**Table 2:** Regional differences in polar bear prey.

	Bearded seal	Harbour seal	Harp seal	Ringed seal	Beluga whale	Walrus	δ^15^N	H-Print
**Baffin Bay**	A	A	A	A	A	A	A	A
**Western Hudson Bay**	B	B	AB	A	B	A	B	A
**Southern Hudson Bay**	AB	B	B	A	B	A	B	A

Pairwise multiple comparisons (Nemenyi’s post-hoc test) to identify where significant between-population differences occur in QFASA estimates of polar bear prey and δ^15^N and H-Print of polar bears. Different letters indicate significant (α = 0.05) differences.

## Discussion

Consistent with previous biomarker studies of marine mammals[29], HBIs were present in all polar bears analysed here. Thus, H-Print data have now been reported across all trophic levels of the Arctic food web, from particulate organic matter[11] to primary and secondary consumers[20, 29, 38] and top trophic level predators here, confirming that the H-Print can be applied throughout the Arctic ecosystem. For polar bears here, this enabled the high contribution of sympagic carbon to diet to be identified (Table 1), revealing the importance of sympagic carbon to the food web, including bears, throughout winter, as hypothesised. Indeed, our data demonstrate that sympagic carbon was underpinning the carbon supply of the majority of bears during sampling (October to May), with comparable H-Print values for both Baffin Bay and Hudson Bay bears, despite the significant regional differences in prey composition (Table 2; Fig 3A).

Diversity of polar bear prey in Baffin Bay and the surrounding regions has been observed previously[33] and is linked to selective foraging related to variability in bear age and sex, sea ice dynamics, migratory patterns and regional differences in prey availability[34] and community structure lower in the food web[39], all of which probably contribute to the variability observed in δ^15^N of bears here (Table 2, Fig 3B). In contrast, neither bear age or sex, nor sampling month or population were significant predictors of polar bear H-Prints here. Further, the generally high proportion of sympagic carbon underpinning polar bear diets during winter did not appear to be prey-dependant. While a larger-scale study of polar bear H-Prints might provide further insight into the reason for the variability in H-Prints observed, we note that estimates of mean sympagic carbon composition were high across the populations studied. Such high sympagic carbon composition is consistent with the high incorporation of sympagic carbon in lower trophic levels, including amphipods[12, 14, 40, 41] and fish[42, 43], which facilitate the transfer of sympagic carbon to higher trophic levels. Our data extend the knowledge of the extent of this transfer by providing numerical estimates of sympagic carbon within polar bears during winter. Indeed, 89% of bears here were sampled over winter (October–March) and all contained IP_25_. Since sympagic carbon (including IP_25_) is only produced within sea ice during the spring sea ice algae bloom (March–June[44, 45]), it is possible that the IP_25_ and related HBIs detected in polar bear livers during winter had bioaccumulated, similar to contaminants such as mercury[46] or organochlorine compounds[47]. If so, H-Print data would be expected to provide an indication of diet averaged over a longer period of feeding (e.g. months to years). However, since turnover times in liver tissue is more rapid than for muscle or adipose[26], for example, we believe H-Print data represent much more recent feeding habits, as demonstrated previously by monthly resolved changes in H-Prints of ringed seals[27]. On this basis we reason that, while the sympagic carbon present in the ecosystem during October to March likely originated from the previous spring bloom (IP_25_ is produced within sea ice during the spring bloom[44]), it only recently became incorporated into the ecosystem. Certainly, following the spring bloom, ice algal cells are exported to the benthos in the Arctic[48–54] resulting in marine sediments containing an important supply of carbon to both pelagic [55, 56] and benthic consumers [57–59] year round. This pathway of sympagic carbon supply to the coastal shelf ecosystem, long after the spring ice algae bloom, has also been observed using HBIs where high abundances of IP_25_ in sediments and benthic consumers, was coupled with an absence of IP_25_ from the overlying waters and sea ice in Rjipfjorden, Svalbard in January 2012[60]. Combined, these observations led to the proposition that sympagic carbon stored in sediments was providing energy to consumers during winter, underpinning the idea that sympagic carbon remains important for the ecosystem, including polar bears, year-round and should, therefore, be considered in conservation assessments.

Assessments of polar bear conservation status are, at present, linked to the impact of sea ice decline as a physical habitat[3], specifically in relation to forcing changes in seasonal sea ice-terrestrial migratory habits [5], hunting and feeding success [6], availability of suitable habitat for denning [7] and the associated effect on the survival of cubs [8]. With each of the regions studied here experiencing recent reductions in sea ice extent and thickness[2, 3], it is anticipated that sympagic carbon availability will also decrease, leading to a likely replacement by pelagic-based systems[61]. Indeed, such change is already becoming evident in Cumberland Sound (south east Baffin Island) where decreasing sea ice extent, coupled with an increased presence of pelagic fish[62], has been proposed as the cause of changing trends in isotope signatures and H-Prints within beluga whale over the last 30 years[29, 63]. However, in contrast to the beluga whale, polar bears are more sensitive to changes in trophic structure and dietary diversity[64] that are likely to result from ongoing decline in sea ice cover[61, 65]. This higher sensitivity to change is supported in the communities studied here where bears are showing reduced body condition that is attributed to sea ice decline in Baffin Bay[66], western Hudson Bay[67], and southern Hudson Bay[68]. As sea ice shows continued decline[69], this may become more pronounced. Indeed, here we observed that bears with the lowest mean sympagic carbon (Baffin Bay), although not statistically significant at present, coincided with the most rapidly increasing summer open water period (Table 1). With a trend of continued sea ice decline[69], it is anticipated that a longer-term H-Print analysis of Baffin Bay polar bears might identify whether this link is significantly related to increasing open water or other factors, such as the presence of the North Water (NOW) polynya in northern Baffin Bay[70]. At present, at least, the high contributions of sympagic carbon observed in polar bears here suggest that future conservation assessments should include estimates of the sympagic/pelagic carbon component of polar bear diet, and this could be especially valuable if applied to long-term monitoring programmes.

## Supporting information

S1 Table Data for individual polar bears. (XLSX)
